# FRED enables standardized FAIR metadata generation and management for omics research

**DOI:** 10.1038/s41598-026-61886-9

**Published:** 2026-07-24

**Authors:** Jasmin Walter, Carsten Kuenne, Noah Knoppik, Philipp Goymann, Mario Looso

**Affiliations:** 1 Bioinformatics Core Unit (BCU), Max Planck Institute for Heart and Lung Research, Bad Nauheim, Germany; 2 Cardio-Pulmonary Institute (CPI), Bad Nauheim, Germany

**Keywords:** Computational biology and bioinformatics, Mathematics and computing

## Abstract

Scientific research relies on transparent dissemination of data and its associated interpretations, including raw data, metadata, experimental design, and data processing details. Production and handling of research data represents an ongoing challenge, extending beyond publication into individual facilities, institutes and research groups, often termed Research Data Management (RDM). It is foundational to scientific discovery and aligned with the FAIR principles. Although the majority of peer-reviewed journals require raw data deposition in public repositories in alignment with FAIR principles, metadata frequently lacks standardization, hindering effective utilization and sharing of research findings. Here we present FRED, a generalized toolkit for FAIR metadata management in omics research based on a flexible, machine-readable YAML format. FRED enables (i) guided, dialog-based creation of metadata files, (ii) structured semantic validation, (iii) logical cross-file search, (iv) API-based integration with external systems, and (v) self-hosted web deployment. We demonstrate the utility of FRED through a complete annotation workflow applied to a published single-nucleus RNA-seq dataset, covering metadata generation, validation, repository-based discovery, and export to NCBI GEO submission format. FRED is designed for non-computational scientists and specialized facilities alike, and integrates into existing RDM infrastructure without requiring dedicated IT resources.

**Supplementary Information:**

The online version contains supplementary material available at 10.1038/s41598-026-61886-9.

## Introduction

Research Data Management (RDM) constitutes a fundamental component of contemporary scientific practice, encompassing the entire lifecycle of data, including sample collection, data generation, organization, storage, access, and dissemination, along with the management of associated metadata. Due to substantial financial and resource expenditures associated with the generation of omics data, maximizing the utility of these datasets through reuse has emerged as a critical objective, thereby driving interest in the integration and repurposing of data from public repositories^1^. However, without proper RDM, experimental designs may be flawed, random and systematic errors can go undetected, and results may be misinterpreted, collectively undermining reliability. In contrast, robust RDM enhances data interpretability and supports independent validation, thereby increasing confidence in research outcomes. Comprehensive metadata provides essential contextual information required for accurate interpretation of data, such as sample origin, experimental conditions, and data processing procedures. It is furthermore mandatory for long-term preservation of scientific knowledge, mitigating the risk of information loss associated with personnel turnover or institutional transitions^2,3^.

The growing emphasis on data-driven science has heightened the demand for standardized, FAIR (Findable, Accessible, Interoperable, Reusable) data practices. Introduced in 2016 by a coalition of industry, academia, and funding agencies, the FAIR principles aim to ensure that scientific data is not only accessible but also semantically rich, machine-readable, and reusable across disciplines and platforms^4^. Achieving this in practice requires both a standardized vocabulary and a structured format capable of capturing the full complexity of omics experimental designs.

Efforts to standardize omics metadata have produced a family of Minimum Information standards: Minimum Information About a Microarray Experiment (MIAME)^3^ for microarrays, Minimum Information about a high-throughput nucleotide SEQuencing Experiment (MINSEQE)^5^ for high-throughput sequencing, and Minimum Information About a Proteomics Experiment (MIAPE)^2^ for proteomics. These standards share a common core of mandatory elements, such as general project information, biological system descriptions, sample sources, experimental variables, and relationships between samples and data, and have been progressively harmonized through initiatives such as Minimum Information for Biological and Biomedical Investigations (MIBBI)^6^, which aggregates minimum information checklists across domains, and the Task Group for Sustainable DwC-MIxS Interoperability^7^, which aligns omics standards with the Darwin Core^8^ biodiversity vocabulary. Tools developed in support of these standards, such as MARMoSET^9^ for proteomics and odMLtables^10^ for neurophysiology, focus primarily on the technical documentation of a measurement. However, they do not provide mechanisms for the guided, flexible capture of experimental design, that is, the structured definition of conditions, experimental factors, and the relationships between samples and conditions. This gap is critical: without it, metadata generation remains dependent on researcher initiative and technical familiarity, limiting consistency and reusability.

Existing approaches for metadata capture and management encompass a wide range of tools with different scopes and purposes. While none of these was designed specifically to solve the problem of standardized experimental metadata, together they represent a landscape of solutions that research groups have to assemble to cover all aspects of this challenge. Centralized database-driven platforms such as openBIS^11^, Yoda^12^, and Coscine^13^ offer powerful infrastructure for data storage, version control, and querying, and can store metadata alongside experimental data. However, their deployment requires substantial technical expertise, dedicated IT infrastructure, and ongoing maintenance, restricting adoption to institutions with dedicated bioinformatics or IT support. File-based standards such as MicroArray Gene Expression Tabular format (MAGE-Tables )^14,15^ and Investigation/Study/Assay Tabular format (ISA-Table )^16^, the latter adopted by FAIR Data Cube^17^, and custom metadata markup languages such as MEtaData Format for Open Reef Data (MEDFORD)^18^ and open metadata Markup Language (odML)^10^ provide lightweight, portable metadata storage with validators and parsers to detect structural and semantic errors. However, they generally do not assist users in the actual creation or editing of metadata files; users must manually construct and maintain these files, a process that is error-prone and inaccessible to researchers without training in data modeling. This problem is partially addressed by tools such as lesSDRF^19^, which provides an interface for creating Sample and Data Relationship Format (SDRF) files, but such tools remain format-specific and do not generalize across omics domains. As a consequence, biomedical laboratories typically capture metadata in paper notebooks, spreadsheets, or electronic lab notebooks (ELNs) initially. These tools are familiar and have a low barrier, but as data analysis progresses, information is frequently re-entered into additional documents, producing fragmented, versioned records that diverge over time and are difficult to reconcile. Importantly, they provide no mechanism for enforcing standardized terminology or structure. Consequently, while each of these approaches addresses part of the problem, no existing solution combines guided, interactive metadata creation with a flexible, file-based architecture and a domain-agnostic vocabulary framework broadly applicable across omics experimental designs.

The importance of standardized terminology becomes especially evident when organizing and storing large amounts of metadata. Inconsistent naming conventions, spelling variations, or the use of synonyms can prevent datasets from being retrieved during searches, significantly limiting their reuse potential. This was demonstrated by the BioSamples Database^1^, which undertook a community-driven curation effort to normalize metadata terminology; resolving spelling differences and harmonizing synonyms increased the number of search results for the term ‘sample type’ by 25%. Such post-hoc curation is, however, labor-intensive and resource-demanding. Embedding controlled terminology at the earliest stages of recording, at the point of metadata creation, is a more efficient and sustainable approach, ensuring greater findability, reusability, and long-term value of omics data. Established biomedical ontologies such as Ontology for Biomedical Investigations (OBI)^20^, Experimental Factor Ontology (EFO)^21^, and the NCBI Taxonomy^22^ provide rich, semantically expressive vocabularies, but their adoption requires ontology engineering expertise that is rarely available in individual research groups or core facilities.

To address these challenges, we introduce the FaiR Experimental Designs (FRED) toolkit for FAIR metadata handling. FRED is based on a machine-readable, YAML-formatted metadata file format designed to be stored alongside experimental data and to fully describe an experiment.

FRED takes a practical approach to controlled vocabulary. Valid terminology is defined through curated YAML-formatted whitelists that can be maintained and extended by domain experts without ontology engineering expertise, and whitelist terms are designed to be compatible with established ontology terms, preserving the option for semantic enrichment where needed.

The toolkit offers guided, dialog-based metadata creation via both a command-line interface and a self-hosted web application, structured semantic validation combining syntactic and logical checks, cross-file metadata search with logical operators, and an application programming interface (API) enabling integration with external systems.

FRED does not replace established minimum information standards such as MIAME, MINSEQE, or MIAPE, nor the tools developed to support them. Rather, it addresses the layer of metadata that precedes and contextualizes what those standards capture: the experimental design itself, comprising the definition of conditions, experimental factors, and the relationships between samples and generated data.

FRED is designed to be complementary to these standards, and supports export to established submission formats including MAGE-TAB (for transcriptomics)^14^ and NCBI Gene Expression Omnibus (GEO)^23^, facilitating downstream data deposition in public repositories.

FRED is fully functional as a standalone tool and can be adopted independently by any research group wishing to begin recording standardized metadata without any prior RDM infrastructure.

At the same time, metadata capture is frequently neglected in institutions where storage-oriented RDM infrastructure already exists. In such cases, data is stored, but its associated experimental context is insufficiently documented. Because FRED’s output is entirely file-based, it can be added to any existing storage structure without requiring migration, reconfiguration, or replacement of the underlying system, filling this gap with minimal integration overhead. This is demonstrated by the integration of FRED into the Cardio-Pulmonary Institute (CPI) repository, where FRED was added to provide structured metadata capture without modification of the underlying system. FRED’s architecture, built around a user-customizable schema file and a modular, extensible whitelist repository, is designed to be accessible to non-computational scientists and requires minimal bioinformatics expertise.

## Results

### Structure, workflow, and features of the FRED toolkit

FRED presents a practical and user-centric solution for the structured generation and management of metadata in omics research. It is implemented in Python and freely available on GitHub at https://github.com/loosolab/FRED or via the Python Package Index (PyPI: https://pypi.org/project/fred-metadata/).

FRED employs a generalized, modular structure (described in the Centralized management section) to define and control supported metadata keys, data types, and validation rules. By default, it supports experimental designs involving *n* distinct conditions defined by experimental factors of interest, such as cell type, genotype, disease stage, enrichment, sex, injury, or tissue.

Individual samples are assigned to these conditions and may represent biological or technical replicates. Each sample can include additional individual or grouped metadata (e.g., age, sex, comorbidities) alongside experimental factors monitored as covariates.

During runtime, FRED dynamically accesses a centralized repository of vocabulary (see Centralized management section below) to **populate interactive dialogs** and **validate input values in real time**, ensuring metadata consistency and avoiding errors due to user inputs.

**Fig. 1:**
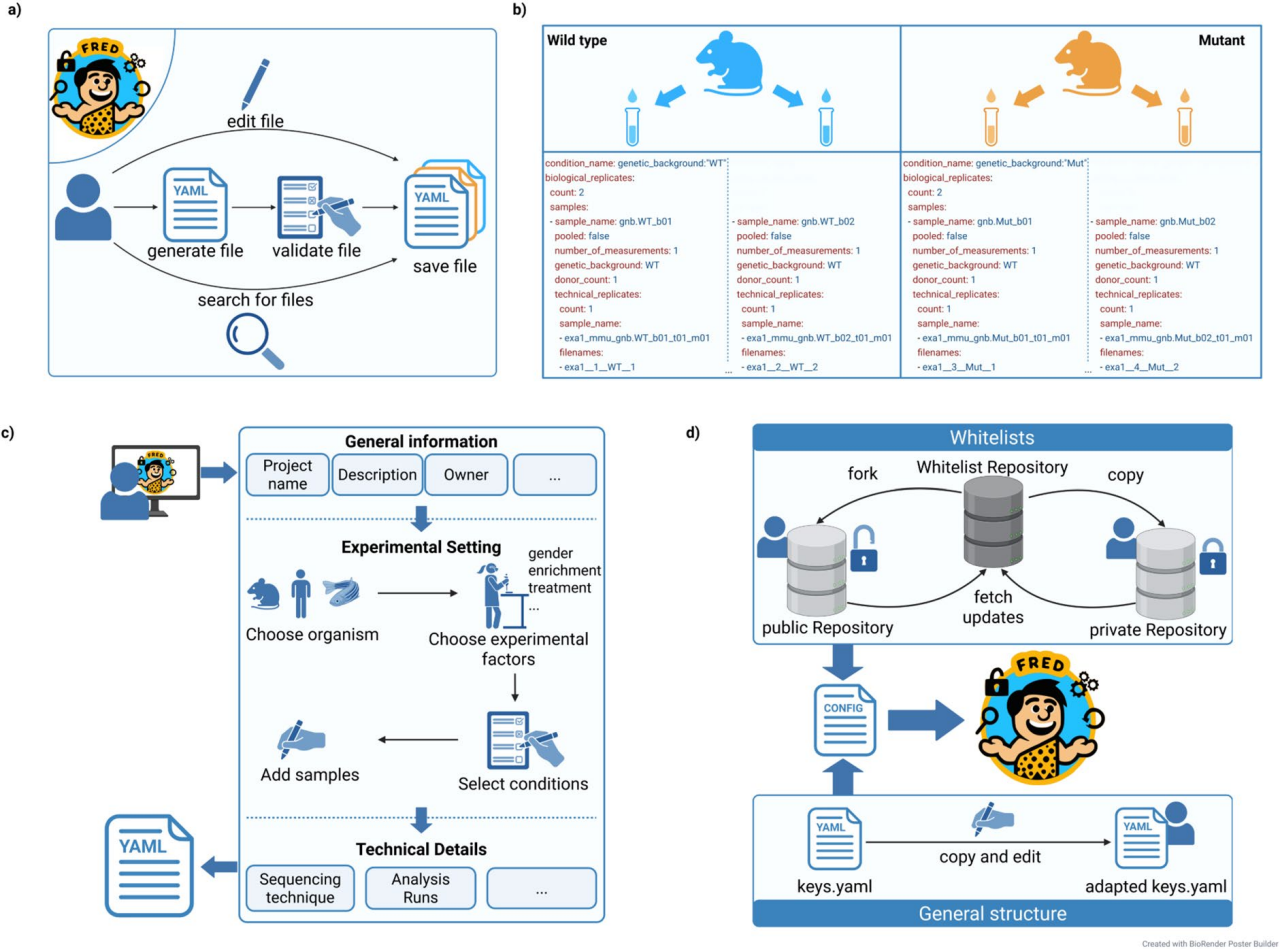
Overview of the FRED toolkit architecture and workflow **a**) The FRED API supports recording, editing, and querying of the metadata stack by a managed vocabulary. **b**) Exemplary structure of a metadata YAML file for an experiment with two conditions. **c**) Workflow for metadata recording, supported by FRED via a command-line or web interface. FRED’s metadata structure is thematically divided into general information about the project, experimental design and technical details. **d**) FRED relies on a whitelist repository and the metadata structure YAML (keys.yaml) to facilitate universal adaptability. Forking or copying the whitelist repository (private or public) and editing of the structure YAML enables adaptation of terms and keys. Created with BioRender.com.

The **workflow of FRED** is characterized by the following sequence of events (Fig. 1a): a user describes an experimental design, this description is recorded in a YAML-formatted file, the file is validated during runtime, and the file collection is stored as a metadata repository locally.

These human- and machine-readable YAML files (Fig. 1b) can be integrated seamlessly into existing RDM structures. This is also true for a wide range of downstream tools and analysis pipelines that rely on the experimental design, since most programming languages offer libraries for reading, writing, parsing, and visualizing YAML files. For example, we integrated FRED into the Cardio-Pulmonary Institute (CPI) repository (https://bioinformatics.mpi-bn.mpg.de/bcu-repository-bn*)*, an RDM structure intended to store all omics experiments generated within the three sites of the excellence cluster project funded by the Deutsche Forschungsgemeinschaft (DFG), and comprising more than 1,000 datasets and metadata files.

To support flexible, generalized, and hierarchical metadata collection, the FRED toolkit provides a guided, incremental dialog for metadata generation via a self-hosted web application (see section below) or command line (Fig. 1c). By guiding users through a structured, intuitive workflow, FRED reduces the risk of missing critical information, promotes consistency across projects, and supports the generation of high-quality, FAIR-compliant metadata from the very beginning of a research study. In addition, FRED automatically generates unique, informative sample IDs and filenames using a standardized format that incorporates project, sample, and experimental information, ensuring each sample is uniquely identifiable and traceable. A comparison of all FRED features with other approaches and solutions developed in the field is shown in Supplementary Table S1.

### Centralized management of valid designs and vocabulary

Metadata structure and whitelist management rely on two code repositories for online/offline/forked usage (Fig. 1d).

All metadata configurations in FRED are centrally managed through a flexible, hierarchical system based on YAML-formatted files. This structure ensures consistency, transparency, and ease of customization across projects and organizations. At the core of the system is the key configuration file ‘keys.yaml’, which defines all permissible metadata keys, their hierarchical placement via indentation, and their structural relationships within the metadata schema.

The metadata schema is thematically organized into three main sections, each represented by a top-level key.

- **project**: Contains general project-level information, including a unique identifier, title, description, and contact details. Contact roles include the data owner, data analyst (if applicable), hosting group, and hosting institution.
- **experimental_settings**: Describes the scientific objectives and structure of the study, specifying how conditions and samples are defined and related. This section allows for multiple experimental setups within one project, enabling the combination of data from different experiments or species.
- **technical_details**: Captures methodological details such as applied omics technologies, processing steps, and other technical aspects, ensuring full traceability and reproducibility.

Each key in the key configuration file is associated with a set of properties that define its behavior within FRED. These properties (summarized in Table 1) include data type (e.g., string, list, integer), whether the field is mandatory or optional, valid value ranges, default values, and validation rules. These settings ensure consistency, prevent invalid entries, and support automated validation during metadata generation.

**Table 1:** Metadata key properties in FRED.

Key	Description
mandatory	Boolean (True/False) indicating whether the field is required.
list	Boolean (True/False) specifying whether the field accepts a list of values.
display name	A human-readable label for the key, shown in the FRED interface.
description	A short explanation of the key’s purpose, displayed to guide users.
value	Default value (e.g., string, number) or null if no default is set.
whitelist	Boolean (True/False) indicating whether valid values are restricted to a predefined whitelist.
input type	Specifies the input method in the interface: select (dropdown), short_text, long_text, bool, number, or date.

FRED employs **whitelists** to enforce standardized user input. Whitelists are stored in a dedicated, publicly accessible repository at https://github.com/loosolab/FRED_whitelists (Fig. 1d). These whitelists are used to restrict input values for string-type fields by providing a predefined set of valid options, thereby preventing inconsistencies due to spelling variations, synonyms, or informal terminology, which are common causes of failed searches or data misinterpretation. FRED’s whitelists adopt taxonomy-based wording for relevant fields such as organism names and gene identifiers, and allow multiple taxonomies to be combined to accommodate diverse experimental contexts.

Users can fork or clone the official whitelist repository to create custom versions or extend existing ones. To maintain alignment with updates from the official repository, GitHub actions are provided that automate the synchronization process. Users can activate these workflows and, if necessary, adapt them to their specific needs, ensuring their local whitelists remain up to date.

Whitelists are categorized into several types to support diverse use cases.

- **plain**: A simple list of valid values for a given key (e.g., a list of tissue types).
- **group**: A dictionary-based structure that organizes longer whitelists into logical categories, improving readability and usability (e.g., grouping diseases by affected organ).
- **depend**: Enables conditional whitelists based on another input. For example, a list of valid gene names depends on the organism (e.g., *Homo sapiens* vs. *Mus musculus*), allowing context-aware validation.
- **abbrev**: Defines abbreviations for values from other whitelists. These abbreviations are used to generate unique, standardized sample file names improving traceability and reducing naming errors.

**Fig. 2:**
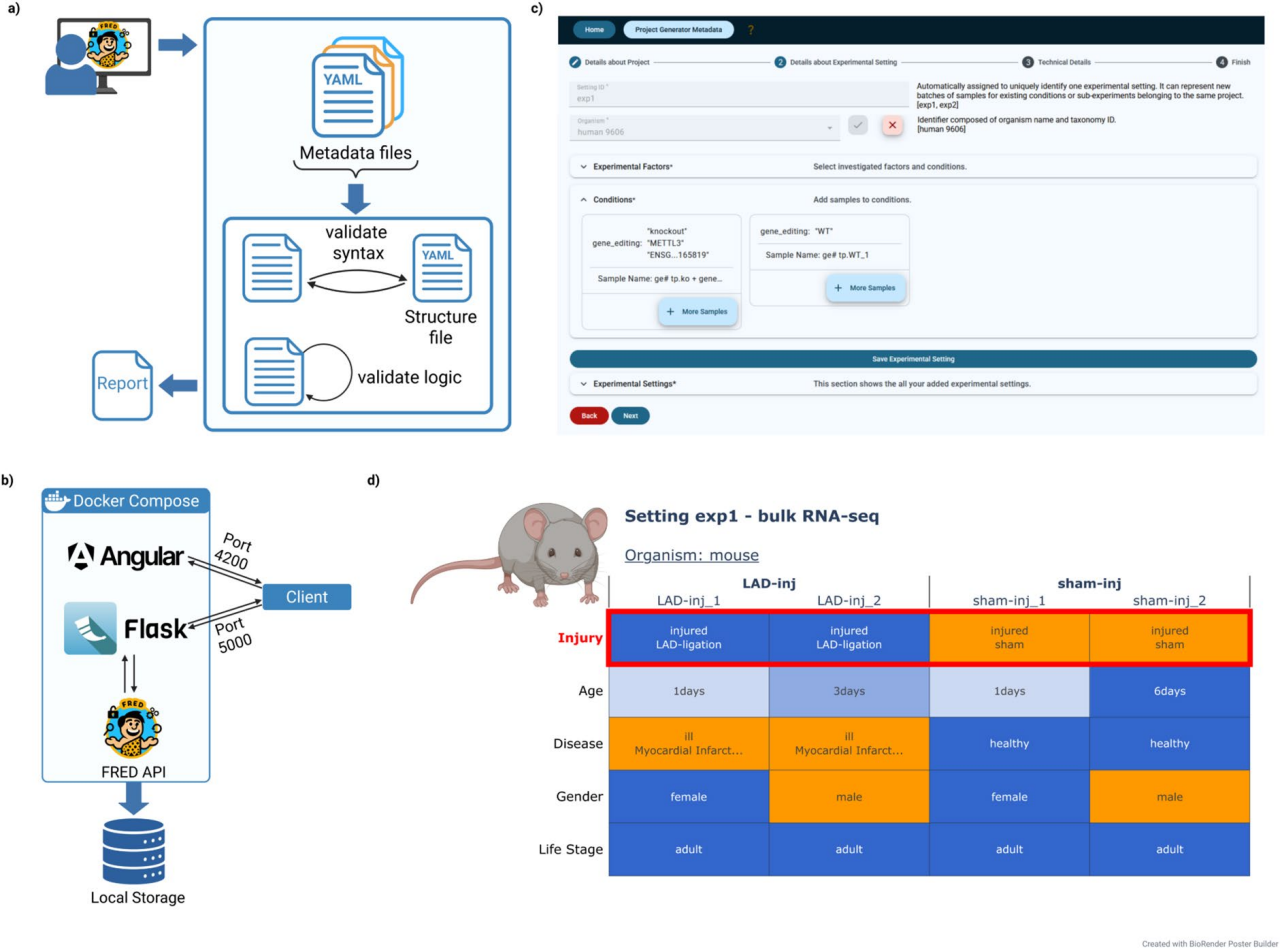
Validation pipeline and user interface features of FRED **a**) FRED provides syntactic and logical validation of metadata files. **b**) The graphical user interface of FRED consists of two containers, a web app using Angular and a REST API using Flask. The containers can be built and started using docker compose. **c**) FRED provides an interface for external calls, e.g. via a web frontend. **d**) FRED provides a summarizing visualization of the experimental design with color-coded values. For quantitative or ordered categorical values (e.g. age, time points, concentrations) FRED uses a continuous color gradient to represent the magnitude of differences. The plot includes information about the organism and the technical method used for sample processing (e.g., RNA-Seq, ChIP-Seq) at the top. This is followed by key experimental factors (e.g., genotype, treatment, disease stage) displayed with a red border. Created with BioRender.com.

FRED regularly performs a validation step on each metadata file, e.g., triggered by a metadata search operation (see below). The validation process itself consists of two phases (Fig. 2a): the **Syntactic Validation and the Logical Validation.** The former ensures that each metadata file conforms to the defined structural schema, including correct hierarchical position of metadata keys within the key configuration file structure, presence of mandatory keys, valid data types, and presence in the corresponding whitelist. In case of syntactic errors, a message is generated listing all deviations from the schema (Supplementary Fig. S2a). Files that fail syntactic validation are excluded from the search to prevent incorrect or misleading results.

During **Logical Validation**, domain-specific consistency checks are performed to ensure that metadata entries are contextually sound. For example, to verify whether the reference genome specified for a sequencing run is compatible with the organism described in the metadata. Logical validation does not affect the file’s syntax, so it does not prevent the file from being processed. Instead, any inconsistencies trigger a warning message that summarizes all logical issues for the user (Supplementary Fig. S2b). These warnings help to identify potential errors or misconfigurations without blocking the search process.

By combining syntactic and logical validation, FRED ensures both structural integrity and semantic coherence of metadata. This dual-layered approach enhances data quality, supports reliable search results, and helps users identify and correct issues early in the data lifecycle, contributing significantly to reproducibility and reusability of omics data.

In summary, the central management system in FRED, combining a structured YAML schema with a modular, extensible whitelist framework, provides a powerful and scalable foundation for standardized metadata collection, iterative structure extension and data integrity. It empowers research teams to maintain high-quality, interoperable data while minimizing errors and supporting long-term data sustainability.

### Guided metadata generation, visualization and search via Web Interface

FRED provides two modes of user interaction. The first method is a command-line interface that can be executed directly on a terminal console. This can be used to generate metadata files via a guided dialog interface or to search for or modify existing files. The second method is designed for users lacking prior experience in computer science or command-line interfaces. Implemented using Angular^24^(v20.3), and deployed locally via Docker^25^ Compose, this self-hosted web application (available at https://github.com/loosolab/FRED_standalone, Fig. 2b) guides users through metadata generation (Fig. 2c), including real-time validation, visualization of experimental designs, and export of metadata in YAML format.

Metadata files can be stored in a central directory and queried via FRED search functions. This approach is designed to enable efficient discovery and retrieval of metadata across multiple projects and experiments. The search requires two mandatory inputs: a root directory for recursive traversal and a search string specifying target metadata values. The search string supports single or multiple values matched against all metadata keys, and can be refined by key-prefixed queries (e.g., organism:“Homo sapiens”) combined via Boolean operators (AND/OR/NOT) with parenthetical precedence control.

FRED includes a built-in function to visualize experimental designs as an interactive plot, enabling users to quickly assess the structure and composition of their experiments (Fig. 2d). Information can be further collapsed to communicate only the minimal study design, and be exported as an interactive HTML or a PNG file.

In summary, by providing a command-line interface as well as a self-hosted web application, FRED ensures broad accessibility across diverse research teams, including biologists, clinicians, and other non-technical users. Its experimental setup visualization routine is, to the best of our knowledge, unique to the field.

### Real-world application: Annotating a published snRNA-seq dataset for the CPI repository

To demonstrate the real-world application of FRED, we annotated a publicly available.

single-nucleus RNA-seq (snRNA-seq) dataset (GEO accession: GSE130699)^26^ hosted in the CPI repository for exploratory analysis via cellxgene. The dataset comprises eight samples from mouse ventricular cardiomyocytes, examining cardiac regeneration potential across two injury conditions (myocardial infarction via Left Anterior Descending (LAD) coronary artery ligation and sham surgery), two developmental stages (postnatal day 1 and postnatal day 8), and two post-operative time points (day 1 and day 3). Without structured metadata, users of this dataset must consult the original publication to identify experimental conditions, replicates, and covariates before any programmatic integration is possible. FRED was applied to capture this information as structured YAML metadata, making it directly explorable within the CPI repository.

**Fig. 3:**
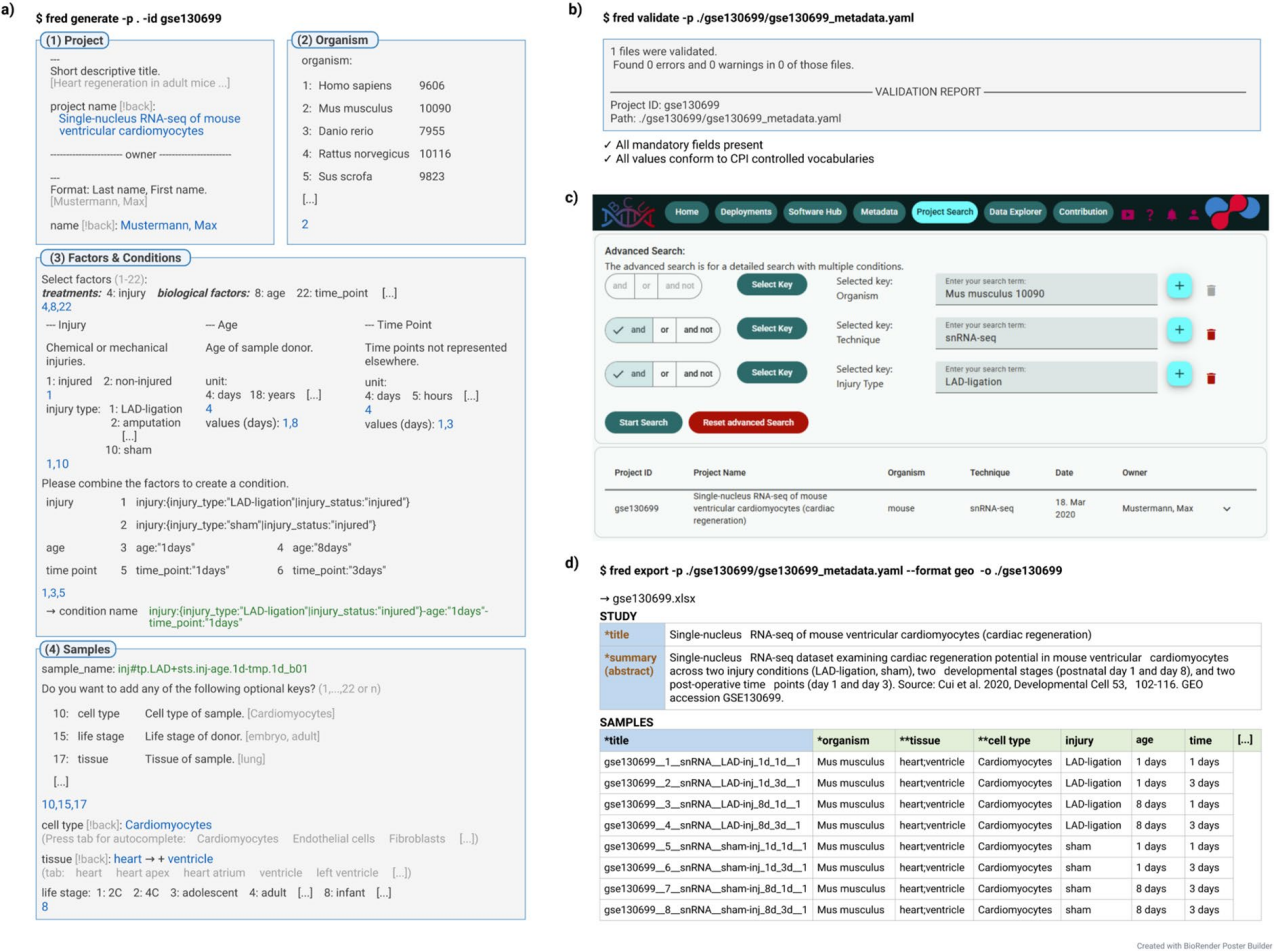
Real-world application of FRED: annotation of a published snRNA-seq dataset **a**) Guided annotation of the publicly available snRNA-seq dataset GSE130699 using the FRED command-line interface (fred generate). The workflow progresses through four steps: (1) project-level metadata including title, date, and owner; (2) organism selection; (3) definition of experimental factors (injury type, age, post-operative time point) and their combination into eight conditions; and (4) sample-level annotation with cell type, tissue, and life stage drawn from CPI-defined controlled-vocabulary whitelists. **b**) FRED validation of the generated GSE130699 metadata file reports zero syntactic errors and zero logical warnings, confirming that all mandatory fields are present, all values conform to CPI-defined controlled vocabularies, and all field combinations are internally consistent across all eight conditions. **c**) Following FRED annotation, the GSE130699 dataset is discoverable through the CPI repository search interface using structured queries combining metadata fields such as organism, technique, and experimental factor values. **d**) FRED exports the validated metadata to NCBI GEO submission format using the fred export command. The resulting Excel file encodes all eight samples with their respective experimental factor values in the structured columns required for GEO deposit. Created with BioRender.com.

Using the FRED command-line interface, we generated a standardized metadata record.

through a single, fully guided interactive session. We followed a four-step annotation workflow (Fig. 3a): (1) providing project-level metadata (title, description, date, owner); (2) specifying the experimental organism (Mus musculus, NCBI taxonomy ID 10090); (3) defining three experimental factors, injury type (LAD-ligation / sham), age (1 day / 8 days), and post-operative time point (1 day / 3 days), and selecting all factor value combinations to yield the eight resulting conditions; and (4) completing sample-level annotations (cell type: Cardiomyocytes; life stage: infant; tissue: heart and ventricle) for each condition from the CPI-defined controlled-vocabulary whitelists. FRED automatically generated standardized filenames and unique, traceable sample identifiers from the factor values (e.g., ‘inj#tp.LAD + sts.inj-age.1d-tmp.1d_b01’), ensuring each sample is unambiguously addressable across downstream analysis pipelines. The complete session comprised 94 interactive steps and produced a structured YAML record (Supplementary File S3).

Structural and semantic validation of the generated YAML was performed immediately after generation using the **‘fred validate’** command. The command reported zero syntactic errors, confirming that all mandatory fields were present and all values conformed to CPI-defined controlled vocabularies, as well as zero logical warnings, verifying that field combinations (e.g., reference genome vs. organism) were internally consistent across all eight conditions (Fig. 3b).

From a researcher’s perspective, the annotated dataset is now directly discoverable through the CPI repository’s search interface without opening the data. A query such as ‘organism: Mus musculus AND technique: snRNA-seq AND injury_type: LAD-ligation’ returns this dataset alongside structurally comparable CPI experiments (Fig. 3c), enabling informed dataset selection prior to any data transfer. This fulfills the Findability and Reusability principles of the FAIR data framework (Table 2).

**Table 2:** FAIR assessment of GSE130699 before and after FRED annotation.

FAIR Principle	Without FRED	With FRED
Findable	Not discoverable via CPI search interface	Searchable by organism, tissue, injury type, age, technique
Accessible	Accessible only via direct link / cellxgene	Machine-readable YAML; API-queryable; pipeline-integrable
Interoperable	No standardized metadata format for tool integration	GEO export; submission-ready Excel file
Reusable	Experimental design only documented in original publication	Fully described with controlled vocabulary; replication-ready

To demonstrate the complete metadata lifecycle, the validated YAML was exported to GEO.

submission format using FRED’s export functionality (Fig. 3d). The resulting Excel file (Supplementary File S4) encodes all eight samples with their respective factor values for injury type, developmental stage, tissue, cell type, and post-operative time point in the structured columns required for NCBI GEO deposit. This illustrates that FRED-annotated metadata can serve both internal repository management and external deposition requirements without duplication of effort, enabling a streamlined path from local data storage to community-standard sharing.

The CPI repository additionally integrates FRED via its Python API, enabling metadata entry through an interactive web-based interface analogous to the self-hosted frontend (see https://loosolab.pages.gwdg.de/software/metadata-organizer/standalone.html#metadata-input for details and an embedded Youtube video^27^.

## Discussion & conclusion

FRED presents a practical, scalable, and user-centric solution for structured generation and management of metadata in omics research, fully aligned with the FAIR principles. The toolkit is designed to support researchers across diverse technical backgrounds, offering both a command-line interface for advanced users and a self-hosted web application. This approach ensures broad accessibility, enabling seamless adoption in laboratories with varying levels of technical expertise.

FRED’s metadata structure is comprehensive and modular, encompassing three essential components: general project information, detailed experimental design and sample annotations, and technical metadata. This structure meets essential requirements for omics research^15,28^, while remaining flexible enough to be applied across other types of high-throughput experiments. Unlike many database-driven systems that require expert knowledge to customize or extend, FRED’s hierarchical, YAML-based design permits straightforward adaptation to evolving projects.

Sample tracking is implemented via automated generation of unique, informative IDs derived from experimental factors and project context. These unique IDs are embedded in the metadata, ensuring traceability of samples and derived files across projects.

FRED stores metadata in YAML format to balance machine-readability with human interpretability. This facilitates integration into automated downstream data analysis pipelines and export to standardized formats including MAGE-TAB and NCBI GEO, as demonstrated in the Real-world Application section above.

The choice of YAML as the underlying metadata format was motivated by several practical considerations. Unlike RDF/OWL or JSON-LD, which require familiarity with semantic web technologies, YAML offers a human-readable syntax that can be inspected and manually verified by researchers without computational training. It is natively supported by libraries in Python, R, Java, and other programming languages widely used in bioinformatics, enabling direct integration into existing analysis pipelines without format conversion. Crucially, the metadata schema itself is defined in a user-customizable configuration file that specifies permissible metadata keys, their hierarchical placement, data types, validation rules, and whitelist associations. Research groups and facilities can extend or adapt this schema to their specific domain requirements without programming expertise, allowing FRED to accommodate diverse experimental designs while maintaining structural consistency. FRED’s whitelist-based validation enforces terminological consistency at input time, compensating for the absence of native semantic relations in YAML such as synonym resolution or subclass hierarchies available in ontology languages. LinkML (Linked Data Modeling Language)^29^ was considered as a simple YAML file alternative during the design of FRED as it defines data models in YAML-based syntax and generates multiple serialization formats including JSON Schema, RDF, and OWL, enabling semantic interoperability with ontology frameworks. However, LinkML’s expressivity comes with a steeper learning curve and requires familiarity with schema definition languages that cannot be assumed for the target users of FRED (non-computational scientists and core facility staff). FRED’s whitelist approach was therefore chosen to prioritize accessibility and local customizability over full semantic expressivity, while remaining compatible with future migration toward LinkML-based schemas. Generating LinkML-compatible schemas or RDF-based exports from FRED metadata would be a candidate for future extension toward broader ontological interoperability. The GWAS Summary Statistics Format (GWAS-SSF)^30^ is similarly pragmatic and pairs a YAML-formatted metadata file with a tab-separated summary statistics data file, providing human- and machine-readable study context for genome-wide association data and serving as the community standard in the GWAS Catalog. FRED follows the same philosophy of combining a lightweight, widely readable format with structured validation to maximize adoption. FRED-generated YAML files can furthermore be included as data entities within a Research Object Crate (RO-Crate)^31^, a community framework for packaging research artifacts together with structured metadata using Schema.org annotations in JSON-LD format. In this configuration, the FRED YAML file provides the experiment-level metadata layer within the RO-Crate bundle, enabling compatibility with RO-Crate-aware repositories and tools. FRED does not currently provide a dedicated export function to automatically generate the required ro-crate-metadata.json descriptor; implementing such an export is a candidate for future development.

Despite its practical advantages, FRED presents several notable limitations. Although guided metadata entry generates YAML files programmatically, thereby eliminating manual indentation errors, users who edit existing files directly must observe YAML’s whitespace-sensitive syntax, necessitating the use of a YAML-aware editor or linting tool. Additionally, while whitelist vocabularies are versioned via repository tags, FRED’s file-based architecture does not provide built-in version control or concurrent-access locking for individual metadata files; research groups requiring these capabilities may complement FRED with a storage platform such as openBIS or Yoda. Furthermore, YAML lacks native support for semantic constructs such as synonym resolution and subclass hierarchies available in ontology languages such as RDF and OWL. While FRED’s whitelist-based approach enforces terminological consistency at input time and is sufficient for structured metadata recording, applications requiring full ontological interoperability would depend on conversion of FRED output to RDF-based formats. The sequential, CLI-driven workflow may also become burdensome for experiments involving numerous conditions or samples. In such cases, users are advised to generate a representative metadata record and repurpose it as a YAML template, thereby minimizing interactive overhead. The web application additionally provides a copy-and-paste function for sample entries, further reducing manual input when annotating multiple similar samples. Notably, FRED saves progress upon completion of each top-level schema section, so interrupted sessions can be resumed from the last saved checkpoint, limiting potential data loss to a single section. Finally, as the number of independent FRED deployments grows, maintaining terminological alignment across independently forked whitelist repositories presents a coordination challenge. While the provided GitHub Actions workflow automates synchronization at the technical level, semantic consistency across forks requires community governance mechanisms that are not yet formalized. Critically, FRED currently provides no functionality to detect divergent definitions of equivalent concepts across deployments, which will require governance structures analogous to those employed by established ontology consortia and constitutes an important direction for future development.

In summary, FRED bridges the gap between rigorous metadata standards and real-world usability. By combining a flexible, extensible architecture with intuitive interfaces and strong support for FAIR principles, it empowers researchers to generate high-quality, reusable metadata. With increasing volume and complexity of omics data, computational tools such as FRED play a critical role in ensuring that data are not only generated but also efficiently documented, discoverable, and reusable.

## Methods

### The program FRED

FRED (v2.0.2) is implemented in Python (v3.12) and hosted on GitHub at https://github.com/loosolab/FRED. It relies on PyYAML (v6.0.2) for reading and writing metadata files and GitPython (v3.1.44) for managing interactions with the whitelist repository. For the generation of interactive visualizations, FRED uses the plotly package (v6.2.0). All dependencies are defined in the pyproject.toml file. The tool, along with all required packages, can be installed using pip in a standalone Python environment with no external dependencies. This ensures FRED runs reliably on any standard Linux system without the need for specialized IT infrastructure. A comprehensive user guide is available at https://loosolab.pages.gwdg.de/software/metadata-organizer/, providing clear instructions for installation, configuration, and usage.

FRED was developed using a requirements-driven, iterative approach without a formal software development framework. The metadata schema and core functionality (guided dialog-based metadata generation, validation, and cross-file search) were defined collaboratively within the team prior to implementation, and development proceeded iteratively in response to evolving requirements. Once the core tool was complete, a dedicated API layer was added to enable integration with the CPI repository, and the codebase was later refactored to improve modularity by restructuring it into classes. Whitelist and schema integrity were validated by continuous integration pipelines from an early stage of development. An automated pytest test suite covering metadata generation, validation, search, export, and web interface functionality was added to complement the CI/CD infrastructure (see Testing and deployment).

### Whitelists

Valid values for metadata fields are stored in a dedicated repository at https://github.com/loosolab/FRED_whitelists in YAML format. To improve performance during runtime, a GitHub Action automatically converts each YAML whitelist into a JSON file. This allows FRED to load and process whitelists more quickly. Users can fork or clone the repository to create custom versions. Pre-configured GitHub Actions enable automatic synchronization with updates from the main repository, ensuring access to the latest standardized terms while maintaining flexibility for local modifications. Whitelist releases are tagged in the repository, enabling users to pin a specific vocabulary version and ensuring that the terminology used for a given set of metadata files remains reproducible over time.

### Docker compose

The standalone version of FRED consists of two containers, a web application using Angular (v20.3) and a REST API using Flask (v2.0.2). The containers can be started with Docker (v28.5) using the command ‘docker compose up’. The standalone version is available at https://github.com/loosolab/FRED_standalone.

### Testing and deployment

FRED is tested by an automated suite comprising 174 unit and integration tests, covering metadata generation, validation, search, export, and web interface functionality (pytest v7.0+, available in the repository under test/). The tool has been in active production use at the Cardio-Pulmonary Institute (CPI) repository since May 2023, where FRED-generated metadata files are created for all new omics projects. As of submission, 599 projects in the CPI repository have been annotated with standardized FRED metadata, demonstrating the tool’s practical utility in an active research data management workflow.

### Figures

Figures were created with BioRender (BioRender.com; RRID: SCR_018361) and assembled using the BioRender PosterBuilder. Publication licenses for individual panels are available at the following URLs: Fig. 1a (https://BioRender.com/f7n3tc3*);* Fig. 1b (https://BioRender.com/xyaep6j*);* Fig. 1c (https://BioRender.com/zisxkpw*);* Fig. 1d (https://BioRender.com/a1uyrbp*);* Fig. 2a (https://BioRender.com/yn4ktgh*);* Fig. 2b (https://BioRender.com/tq92tz0*);* Fig. 2c (https://BioRender.com/k6j1j90*);* Fig. 2d (https://BioRender.com/wpqp9ry*);* Fig. 3a (https://BioRender.com/2knlwpd*);* Fig. 3b (https://BioRender.com/t5xmnuq*);* Fig. 3c (https://BioRender.com/d9airkm*);* Fig. 3d (https://BioRender.com/hgizzmy*);* Supplementary Fig. S2a (https://BioRender.com/necnl2g*);* Supplementary Fig. S2b (https://BioRender.com/ikrvpmy*).*

## Supplementary Information

Below is the link to the electronic supplementary material.

Supplementary Material 1

Supplementary Material 2

Supplementary Material 3

Supplementary Material 4

## Data Availability

No datasets were generated or analysed during the current study. All code described, exemplary files as generated, and respective docker images are available via github https://github.com/loosolab/FRED , https://github.com/loosolab/FRED_standalone / , https://github.com/loosolab/FRED_whitelists.

## Code availability

FRED (v2.0.2) is freely available under the MIT license at https://github.com/loosolab/FRED and via the Python Package Index (PyPI) as fred-metadata. A persistent, citable release is available at https://doi.org/10.5281/zenodo.20595412. Machine-readable citation metadata is provided in the repository as CITATION.cff. Controlled vocabulary whitelists are maintained in a companion repository at https://github.com/loosolab/FRED_whitelists (MIT license). The self-hosted web frontend is available at https://github.com/loosolab/FRED_standalone (MIT license). Comprehensive user documentation is available at https://loosolab.pages.gwdg.de/software/metadata-organizer/. The complete YAML metadata record generated for the GSE130699 real-world application (Supplementary File S3) and the corresponding GEO submission Excel file (Supplementary File S4) are provided as supplementary data accompanying this article. Example metadata files are additionally available in the FRED repository under /examples/.
